# Towards high-throughput molecular detection of *Plasmodium*: new approaches and molecular markers

**DOI:** 10.1186/1475-2875-8-86

**Published:** 2009-04-29

**Authors:** Nicolas Steenkeste, Sandra Incardona, Sophy Chy, Linda Duval, Marie-Thérèse Ekala, Pharath Lim, Sean Hewitt, Tho Sochantha, Doung Socheat, Christophe Rogier, Odile Mercereau-Puijalon, Thierry Fandeur, Frédéric Ariey

**Affiliations:** 1 Institut Pasteur du Cambodge, Laboratoire d'Epidémiologie Moléculaire, BP 983, Phnom Penh, Cambodia; 2 Institut Pasteur, Unité de Pathogénie Virale, Paris, France; 3 Institut Pasteur, Unité d'Immunologie Moléculaire des Parasites, CNRS URA 2581, Paris, France; 4 European Commission National Malaria Control Program, Phnom Penh, Cambodia; 5 National Center for Parasitology, Entomology and Malaria Control, Phnom Penh, Cambodia; 6 Institut de Recherche Biomédicale des Armées – Antenne de Marseille/IMTSSA, Unité de Recherche en Biologie et Epidémiologie Parasitaires – Equipe «Moustiques et Maladies Emergentes » – UMR 6236 – URMITE, Marseille, France

## Abstract

**Background:**

Several strategies are currently deployed in many countries in the tropics to strengthen malaria control toward malaria elimination. To measure the impact of any intervention, there is a need to detect malaria properly. Mostly, decisions still rely on microscopy diagnosis. But sensitive diagnosis tools enabling to deal with a large number of samples are needed. The molecular detection approach offers a much higher sensitivity, and the flexibility to be automated and upgraded.

**Methods:**

Two new molecular methods were developed: dot18S, a *Plasmodium*-specific nested PCR based on the *18S rRNA *gene followed by dot-blot detection of species by using species-specific probes and CYTB, a *Plasmodium*-specific nested PCR based on *cytochrome b *gene followed by species detection using SNP analysis. The results were compared to those obtained with microscopic examination and the "standard" *18S rRNA *gene based nested PCR using species specific primers. 337 samples were diagnosed.

**Results:**

Compared to the microscopy the three molecular methods were more sensitive, greatly increasing the estimated prevalence of *Plasmodium *infection, including *P. malariae *and *P. ovale*. A high rate of mixed infections was uncovered with about one third of the villagers infected with more than one malaria parasite species. Dot18S and CYTB sensitivity outranged the "standard" nested PCR method, CYTB being the most sensitive. As a consequence, compared to the "standard" nested PCR method for the detection of *Plasmodium spp*., the sensitivity of dot18S and CYTB was respectively 95.3% and 97.3%. Consistent detection of *Plasmodium spp*. by the three molecular methods was obtained for 83% of tested isolates. Contradictory results were mostly related to detection of *Plasmodium malariae *and *Plasmodium ovale *in mixed infections, due to an "all-or-none" detection effect at low-level parasitaemia.

**Conclusion:**

A large reservoir of asymptomatic infections was uncovered using the molecular methods. Dot18S and CYTB, the new methods reported herein are highly sensitive, allow parasite DNA extraction as well as genus- and species-specific diagnosis of several hundreds of samples, and are amenable to high-throughput scaling up for larger sample sizes. Such methods provide novel information on malaria prevalence and epidemiology and are suited for active malaria detection. The usefulness of such sensitive malaria diagnosis tools, especially in low endemic areas where eradication plans are now on-going, is discussed in this paper.

## Background

The first methods for molecular detection of *Plasmodium falciparum *malaria were published in the 1980s [1-3]. Since then, much progress has been made in DNA extraction, and detection protocols have been simplified. The four human species can be specifically identified, and the more recently developed real-time amplification techniques allow rapid processing of samples and quantification of parasite loads [4-7]. Molecular assessment of point prevalence at the village level has also become feasible. Various field surveys have shown that molecular methods detected up to eight times more *Plasmodium spp*. infections than microscopy, and that mixed infections could represent up to one third of them [8-11]. Molecular detection tools modify the interpretation of malaria epidemiology, by revealing large reservoirs of asymptomatic infections [8,12,13], cryptic species potentially influencing transmission patterns and clinical outcomes [14,15], as well as shifts in age distribution of *Plasmodium spp*. infections [16]. The main drawback of molecular detection tools is their cost and workload. Furthermore none of the published methods is adapted to large-scale analysis of thousands of samples. Therefore, most decisions for malaria control programmes still rely on data collected by health services, where malaria is diagnosed using microscopy.

With recent moves towards malaria elimination in a number of countries [17,18], additional methods for detecting infections and infectious reservoirs are needed. Indeed, large-scale studies will be required, including in areas with difficult access to health centers. Such studies require high sensitivity in order to detect asymptomatic carriage and improved species identification in order to adapt treatment. Such needs are best fulfilled by molecular methods, which furthermore would open the possibility to monitor vector control measures. The aim of this study was to improve molecular detection of the four *Plasmodium *species. To this end, new molecular detection approaches were developed targeting the nuclear *18S rRNA *gene (*18S rDNA*), and the mitochondrial cytochrome *b *(*cytb*) gene, enabling mass screening of field samples for epidemiological studies. Both loci were chosen because of their obligate presence, their good intra- species conservation associated with appropriate inter-species variation. The methods were validated using laboratory isolates and used to explore point prevalence in field samples collected during a cross-sectional prevalence survey in three villages in Rattanakiri Province, Cambodia [19]. Results were compared with standard detection methods: microscopy and a published nested PCR method [6], in order to evaluate their performance in terms of sensitivity, specificity and suitability for epidemiological studies.

## Methods

### Study sites

The three study villages are located in the north-eastern province of Rattanakiri in Cambodia. Rattanakiri is a hilly and forested province, which borders Laos and Vietnam. It is considered one of the most malaria endemic areas in the country. The principal vectors are *Anopheles dirus*, *Anopheles maculatus *and *Anopheles minimus *[20,21]. In 2001, the reported incidence of malaria cases based on laboratory microscopy diagnosis was 21 per 1,000 inhabitants, of which 79.6% and 20.4% were due to *Plasmodium falciparum *and *Plasmodium vivax *infections, respectively [22].

In September 2001, a baseline cross-sectional prevalence survey conducted by the European Commission-Cambodia Malaria Control Programme (ECMCP) team [19] included 36 "high-risk" villages (less than 1 km from the forest), which had never been included in any bed net distribution project. Subjects were chosen by random selection of households. Fingerprick blood was collected, and thin/thick blood smears, as well as double blood-spots (approximately 20 μL of blood on Whatman 3 M filter paper) were prepared. The blood spots were stored at -20°C until analysis. The study reported here concerned three villages: the village of Ping was chosen because it had shown the highest malaria prevalence by microscopy diagnosis. The villages Pahoy and Smach were randomly selected from the list of the 36 ECMCP villages.

### Ethical approval

The study was approved by the National Ethics Committee of the Kingdom of Cambodia, and all participants gave informed consent.

### Microscopy

Thin smears were fixed in methanol. Both thin and thick smears were stained with 3% Giemsa for 30 minutes at room temperature. Examination was performed by experienced microscopists at the National Centre for Parasitology, Entomology and Malaria Control in Phnom Penh. At least 100 thick film fields with 1,000× magnification were examined before a slide was considered negative. Malaria case definition was based on the presence or absence of *Plasmodium *parasites on microscopy slides. Parasite species and stages were confirmed on the thin film. No second reading was possible.

### Samples for sensitivity and specificity studies

For evaluation of DNA extraction methods, cultured *P. falciparum *3D7 parasites at 1% parasitaemia were serially diluted ten-fold using uninfected blood. Aliquots of 20 μl were spotted on Whatman 3 M filter paper, air-dried and stored at -20°C.

For evaluation of the sensitivity of the molecular detection methods, a 200 μl red blood cell pellet of a 7.5% *P. falciparum *3D7 culture was extracted by the QIAamp DNA blood kit (QIAGEN, Germany). DNA eluted with 200 μl of water was stored at -20°C until analysis. For sensitivity studies, the DNA was serially diluted ten-fold and used for PCR amplification.

The specificity of the molecular diagnosis methods has been assessed with reference DNA of the four species infecting human. DNA from *P. falciparum *was extracted from a continuous culture of the 3D7 strain, using the QIAamp DNA Blood Mini kit (QIAGEN, Germany). DNA from the *P. vivax *Belem strain was kindly provided by Peter David (Institut Pasteur de Paris, France). *Plasmodium malariae *and *Plasmodium ovale *DNA as well as control human DNA from a non-infected person were kindly provided by Georges Snounou (Muséum d'Histoire Naturelle, Paris, France). DNA from patients with *P. malariae *and *P. ovale *single infections, were kindly provided by Eric Legrand (Institut Pasteur de Cayenne, French Guiana). Species identification and single infection were confirmed by *cox1 *and *cytochrome b *genes [23] sequencing and by the "standard" PCR (see below).

### DNA extraction

The blood spots collected in Ping village were processed by 96 well plate extraction method using the QIAamp DNA blood 96 kit (QIAGEN, Germany) as described previously [24]. Cut-out blood-spots were transferred in 96 well plates adapted for 1 mL volumes, incubated for 90 minutes at 4°C in 1 mL buffer HBS (HEPES 10 mM, NaCl 140 mM, KCl 10 mM) with 0.5% v/v saponin (SIGMA Aldrich, Germany), then washed twice with 1 mL HBS using a vacuum pump and a manifold. Subsequently, the recommendations of the kit supplier were followed. Briefly, filter papers were treated for 10 minutes with 180 μL buffer ATL at 85°C, then digested with 4 μl of proteinase K at 100 mg/mL for 1 hour at 56°C. After addition of 200 μL buffer AL and incubation for 10 more minutes, filter papers were spun down; the supernatant was recovered and mixed with 200 μL 100% ethanol (SIGMA Aldrich, Germany) at room temperature. The mixture was filtered through QIAamp DNA blood columns using a vacuum pump, then washed once with 500 μL buffer AW1 and twice with 500 μL buffer AW2. DNA was eluted with 100 μL buffer AE and stored at -20°C.

DNA from the blood spots of the Pahoy and Smach villages was extracted with the Instagene resin (BIORAD, USA) and a 96 well plate in-house protocol adapted from the suppliers recommendations. First, the above described red blood cell lysis in HBS/saponin and the wash steps in HBS were performed. Then, 200 μL preheated Instagene resin was added to the filter papers, the mixture incubated for 30 minutes at 56°C, briefly vortexed, and incubated at 90°C for another 30 minutes. After centrifugation at a minimal speed of 4,000 rpm for 20 minutes, the supernatant was recovered and stored at -20°C.

### *18S rDNA*: species-specific nested PCR, "standard PCR" (Std)

The nested PCR method based on the *18S rRNA *gene marker [7] adapted for epidemiological studies [6] was considered as reference. After a *Plasmodium spp*. specific nested PCR with the primer pairs rPLU1/5 and rPLU3/4, the primary PCR product of *Plasmodium *positive samples was separately amplified with the four species-specific primer pairs rFAL1/2, rVIV1/2, rMAL1/2, rOVA1/2 to identify the species. The published protocol was strictly followed, by using 4 μL of DNA and 2 μL of primary PCR product for the primary and secondary PCR reactions, respectively. Detection was done by 2% agarose gel electrophoresis and ethidium bromide staining.

### *18S rDNA*: Genus-specific nested PCR and species-specific dot blot detection (dot18S)

Selection of primers and probes for this new detection approach was based on alignment of *18S rDNA *sequences of human and primate *Plasmodium *species (Table 1), with primers and genus-specific probes being chosen in zones of maximal sequence similarity of all *Plasmodium *species, but not with the 18S rDNA of other *Apicomplexa *and human. The V7 variable zone was targeted because of its short length, significant differences between species, but good similarity of stage-specific copies within each species. The choice of the species-specific probes was based on the intention to detect all stage-specific sequences of the same species. For each primer and probe, two to four different designs were experimentally evaluated, using the above-mentioned reference DNA samples, before making a final choice for highest possible sensitivity and specificity.

**Table 1:** Primers and probes used for Dot-blot *18S rDNA*-based detection and primers for *cytochrome b*-based detection

	5' – 3' sequences	specificity
**Dot-blot *18S rDNA*-based detection**		
**Primary PCR**		
Ps	CTT TCT TgA TTT CTT ggA	*P. spp*.
PPas	ATT CCT CgT TCA AgA TTA A	*P. spp*.
**Nested PCR**		
Ns1	CAT ggC CgT TTT Tag TTC gTg AAT AT	*P. spp*.
Nas new	CAC gCg TgC AgC CTA gTT	*P. spp*.
**Probes**		
PLAS1	ATA ACg AAC gAg ATC TTA ACC	*P. spp*.
FAL	CTC TAT TTC TCT CTT CTT TTA AgA	*Pf *type A, S
VIVA1	AAT ATT ggg ATA CgT AAC AgT	*Pv *type A
VIVS1	gTT TCT TAA TCg AAT AgC TgA	*Pv *type S
MAL2b	AgA ATA TAg ATA AAT TgT gCT AA	*Pm*
OVA3	TgA AAT TgA ATA TAg CTg AAT T	*Po*

***Cytochrome b*-based detection**		

**Primary PCR**		
GCDW2	Cgg TCg CgT CCg gTA gCg TCT AAT gCC TAg ACg TAT TCC TgA TTA TCC Ag	*P. spp*.
GCDW4	CgC ATC ACC TCT ggg CCg CgT gTT TgC TTg ggA gCT gTA ATC ATA ATg Tg	*P. spp*.
**Nested PCR**		
PLAS1	gAg AAT TAT ggA gTg gAT ggT g	*P. spp*.
PLAS2	Tgg TAA TTg ACA TCC AAT CC	*P. spp*.

Specificity of primers and probes is indicated as follows: *P. spp*. = *Plasmodium spp*., *Pf*/*Pv*/*Pm*/*Po *= *P. falciparum*, *P. vivax*, *P. malariae *and *P. ovale*, respectively, type A/S = stage-specific *18S rDNA *type A and S, respectively.

The primary PCR was performed in a 20 μL volume with 3 μL DNA, 0.4 μM of Ps and PPas primers (Table 1), 0.2 mM dNTP, 1.5 mM MgCl_2_, and 1 U of Taq DNA polymerase (Solis Biodyne, Estonia), using the following cycling program: 4 minutes at 95°C, then 30 cycles of 30 seconds at 95°C, 90 seconds at 51°C, 2 minutes at 64°C, and final extension for 15 minutes at 64°C. One μL of primary PCR product was used for nested PCR under the same conditions, using the primers Ns1 and Nas new (Table 1), except for the annealing (60°C) and extension (66°C) temperatures, and the number of cycles (25).

The colorimetric Dot-blot detection was designed for a 96 well plate format, using the Bio-Dot Microfiltration apparatus (BIORAD, USA). The protocol was adapted from the suppliers recommendations. Briefly, 10 μL of nested PCR product were denatured 15 minutes at 40°C in 0.2 M NaOH, then transferred to the Bio-Dot apparatus mounted with a TE (Tris 10 mM, EDTA 1 mM, pH 8.0) soaked Hybond N+ membrane (Amersham, UK). After successive filtration of the PCR product and 500 μL 0.2 M NaOH using a vacuum pump, the membrane was rinsed in SSC 2× (SSC 20×: NaCl 3 M, Na citrate 0.3 M, pH 7.0), air-dried and exposed for 5 minutes to UV light for DNA crosslinking. The subsequent steps were performed in sealable hybridization bags (Roche Diagnostics, France) and a waterbath. After 2 hours pre-hybridization at 40°C in Denhardt's hybridization buffer (SSC 5×, 0.1% Ficoll, 0.1% polyvinylpyrrolidone, 0.1% bovine serum albumine, 0.5% SDS), 10 pmol/mL of digoxigenine-labeled probe (PROLIGO, France) was added and incubation continued overnight or 3 hours minimum (probes sequences: Table 1).

Wash steps were as follows : two times 5 minutes in SSC 2×, SDS 0.1% at room temperature, 15 minutes in SSC 1×, SDS 0.1% at 40°C, and two times 10 minutes in SSC 0.1×, SDS 0.1% at 40°C. For the revelation, the membrane was incubated for 5 minutes in "Washing buffer" and for 1 hour in "Blocking Solution" (DIG Wash and Block buffer set, Roche Diagnostics, France) at room temperature, then 30 minutes in "Blocking Solution" with 1,000× diluted anti-digoxigenine antibody conjugated to alkaline phosphatase (Roche Diagnostics, France), then 15 minutes in "Washing Solution" and 3 minutes in "Detection buffer". Colorimetric revelation was achieved with 50× diluted NBT/BCIP in "Detection buffer" (NBT/BCIP stock solution, Roche Diagnostics, France). After having obtained a satisfying signal intensity, reaction was stopped by rinsing in TE buffer, and the membrane was scanned in a GS 800 densitometer (BIORAD, USA).

Sequences of *Plasmodium *genus-specific primers for nested PCR amplification and probes for Dot-blot colorimetric detection on membranes were selected after alignment of the following *18S rDNA *sequences: *P. falciparum*: M19172, M19173; *P. vivax*: U07367, U93234, AF145335, U07368, U93095; *P. malariae*: M54897; *P. ovale*: L48986, AJ001527; *Plasmodium simium*: U69605; *Plasmodium fragile*: M61722; *Plasmodium knowlesi*: U83876; *Plasmodium cynomolgi*: L08241; *Plasmodium reichenowi*: Z25819; *Plasmodium inui*: U72541; *Plasmodium brasilianum*: AF130735; *Toxoplasma gondii*: L37415; human: X03205.

### *Cytochrome b*: genus-specific nested PCR and SNP identification (CYTB)

**Figure 1:**
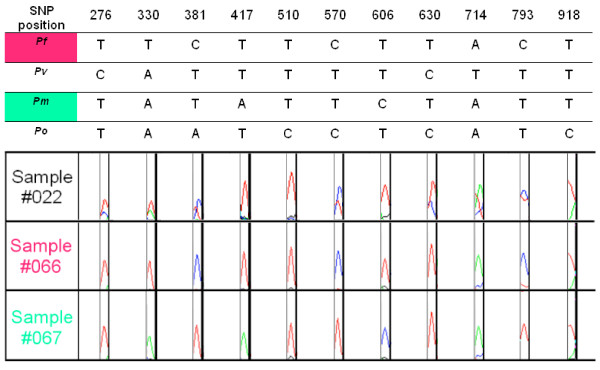
**Species-specific combinations of SNP in the *cytochrome b *gene**. 11 SNPs were selected allowing categorical identification of the *Plasmodium *species. Three examples of SNP sequencing data are shown: sample #22 = mixed infection of *P. falciparum *and *P. vivax*, sample #66 = *P. falciparum*, and sample #67 = *P. malariae *(Seqscape 2.0 software, Applied Biosystems USA). Those SNPs were shown to be robust and never presented contradictory results. *Pf*/*Pv*/*Pm*/*Po *= *P. falciparum*, *P. vivax*, *P. malariae *and *P. ovale*, respectively.

Two primer sets were designed for nested PCR amplification of a large portion of the *cytochrome b *gene, with specificity to all human and primate *Plasmodium *species, but excluding other *Apicomplexa *or human DNA. Primers are presented in Table 1[23,25]. One μL DNA was amplified with 1 μM of each primer, 0.2 mM dNTP (Solis Biodyne, Estonia), 3 mM MgCl_2_, and 2 U Taq DNA polymerase (Solis Biodyne, Estonia), using the following cycling program : 5 minutes at 94°C, then 40 cycles of 30 seconds at 94°C, 90 seconds at 60°C, 90 seconds at 72°C, and final extension 10 minutes at 72°C. For the nested-PCR, 2 μL of primary PCR product were amplified under the same conditions, except for the MgCl_2 _concentration (2.5 mM). PCR products of 815 base pairs were detected by standard 2% agarose gel electrophoresis and ethidium bromide staining. Double strand sequencing of PCR products was performed by the Genopole laboratory of the Institute Pasteur of Paris. Sequences were analysed with the Seqscape 2.0 software (Applied Biosystems, USA) to identify species-specific SNP combinations. An algorithm based on 11 selected SNPs has previously been developed based on an alignment of published *cytochrome b *reference sequences of *P. falciparum*, *P. vivax*, *P. malariae *and *P. ovale*, allowing identification of each species (Figure 1), as well as all possible combinations of mixed infections.

### Statistical analysis

All statistical analyses were performed by using STATA SE 8 software (Stata Corporation, College Station, TX). Exact McNemar test has been used to compare frequencies in matched data of infections.

## Results

### Comparison of DNA extraction methods

To compare the QIAamp 96 and Instagene 96 DNA extraction methods, filter paper blood spots of ten-fold serially diluted blood (ranging from 1% to 10^-5^% parasitaemia) were extracted in parallel using these two methods. DNA was amplified using the standard nested PCR method targeting *18S rDNA*. Both methods allowed repeated detection of 10^-4 ^dilutions, whereas the 10^-5 ^dilutions were only detected in one of two experiments. This "all or none" phenomenon at detection limit has been reported previously [6,7]. The QIAamp 96 protocol was discarded because of sporadic foaming during the washing steps (increasing the risk of cross-contamination of flanking wells), the higher workload and more expensive kits, compared to the Instagene 96 method.

### Screening of field samples by microscopy and "standard" nested PCR

A total number of 337 samples were screened in the three villages. Microscopy diagnosis revealed a *Plasmodium *prevalence of 59.8%, 39.6% and 29.1% in Ping (n = 102), Smach (n = 101), and Pahoy (n = 134), respectively. The infections consisted in *P. falciparum *(prevalence of 48.0%, 33.7 and 28.4%, respectively), *P. vivax *(2.0%, 1.0% and 0.7%, respectively) or a mixed infection of both *P. falciparum *and *P. vivax *(9.8%, 5.0% and 0%, respectively).

**Figure 2:**
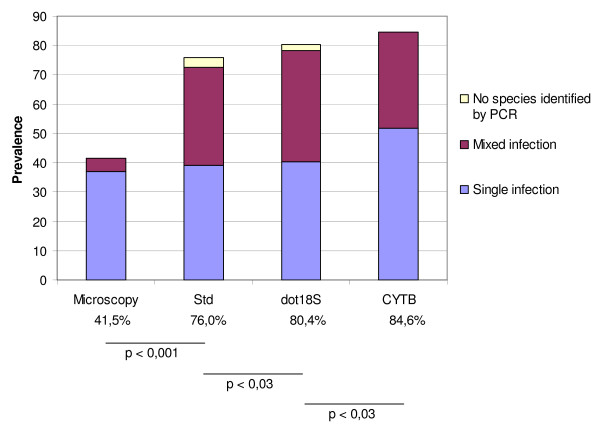
***Plasmodium *prevalence measured by microscopy and molecular methods**. Significant (exact McNemar tests) differences in prevalence rates of *Plasmodium spp*. infections (n = 337) were detected by microscopy, "standard" nested PCR (Std), *18S rDNA*-based nested PCR and Dot-blot detection (dot18S), and by *cytochrome b*-based nested PCR and SNP identification (CYTB). The proportion of mixed infections detected by the molecular methods was also considerably higher than by microscopy (43.7% for Std, 47.2% for dot18S, and 38.9% for CYTB, compared to 10.7% for microscopy).

The published "standard" PCR detected significantly (exact McNemar test, p < 0.0001) more *Plasmodium spp*. infections than microscopy in all three villages (Figure 2). Table 2 shows the comparison with microscopy for the village Ping, which had the highest microscopy-based malaria prevalence of all 36 initially surveyed villages (Table 2), and for Smach and Pahoy villages (Table 2). The data show that molecular detection multiplied the estimated *Plasmodium spp*. prevalence by 1.3 in Ping, and by 2.2 in Smach and Pahoy. *P. malariae *and *P. ovale *were detected in the three villages with a prevalence of 15.1% (range 12.7%–15.7%) and 6.5% (range 3.9%–7.9%), respectively, although these species were considered rare or non-existent in Cambodia and had not been detected in these samples by microscopy. Moreover, the PCR approach revealed a much higher rate of mixed infections (39% and 31% prevalence in Ping and Smach/Pahoy, respectively) than microscopy, with numerous triple or quadruple infections.

**Table 2:** Comparison of malaria diagnosis by microscopy and "standard" nested PCR The comparison between microscopy and "standard" PCR diagnosis is shown for samples collected in the Ping village (n = 102, Table 2A) and for samples collected in the Smach and Pahoy villages (n = 235, Table 2B)

	Standard PCR															(%)
Microscopy	Neg	*Pf*	*Pv*			*Pf*+*Pv*	*Pf*+*Pm*	*Pf*+*Po*			*Pf*+*Pv*+*Po*	*Pf*+*Pv*+*Pm*	*Pf*+*Pv*+*Pm*+*Po*	Total		

Neg	20	13				7	1							41	neg	(40.2)
*Pf*	4	20	1			10	6	1				6	1	49	si	(50.0)
*Pv*			1			1								2		
*Pf*+*Pv*	1		2			4					1	1	1	10	mi	(9.8)
Total	25	33	4			22	7	1			1	7	2	102		

	neg	si				mi										
	
(%)	(24.5)	(36.3)				(39.2)										

	Standard PCR															(%)

Microscopy	neg	*Pf*	*Pv*	*Pm*	*Po*	*Pf*+*Pv*	*Pf*+*Pm*	*Pf*+*Po*	*Pv*+*Pm*	*Pv*+*Po*	*Pf*+*Pv*+*Po*	*Pf*+*Pv*+*Pm*	*Pf*+*Pv*+*Pm*+*Po*	Total		

Neg	61	47	10	3	2	14	4	2	2	1	1	7	2	156	neg	(66.4)
*Pf*	7	26	5			15	4	1	1		4	6	3	72	si	(31.5)
*Pv*		1	1											2		
*Pf*+*Pv*						1					1	2	1	5	mi	(2.1)
Total	68	74	16	3	2	30	8	3	3	1	6	15	6	235		

	neg	si				mi										
	
(%)	(28.9)	(40.4)				(30.6)										

*Pf*/*Pv*/*Pm*/*Po *= *P. falciparum*, *P. vivax*, *P. malariae *and *P. ovale*; neg = negative; si = single infection; mi = mixed infection

A total of 12 samples were considered microscopy positive, but tested negative by "standard" nested PCR (Std). Out of these, eight samples were subsequently found to be *Plasmodium *positive by at least one of the newly developed and more sensitive molecular detection methods (Dot18S and CYTB, see following paragraph). All four remaining samples displayed low parasite densities by microscopy (rating of 1+).

### Comparison of molecular detection approaches for genus (*Plasmodium *spp.) detection

The sensitivity of the three molecular detection methods was assessed by two experiments with ten-fold serially diluted DNA samples of known parasite content (corresponding to a parasitaemia range from 750,000 down to 0.0075 parasites per μL blood, p/μL). The "standard" PCR detected down to 7.5 p/μL, with an "all or none" phenomenon for 0.75 p/μL. Higher sensitivity was obtained with dot 18S and CYTB methods, which detected down to 0.75 p/μL and 0.075 p/μL, respectively.

The estimated prevalence rate of plasmodial infections was significantly higher with CYTB than with dot 18S (exact McNemar test, p < 0.03) and significantly higher with the dot 18S than the "standard" nested PCR method (p < 0.03) (Figure 2).

**Figure 3:**
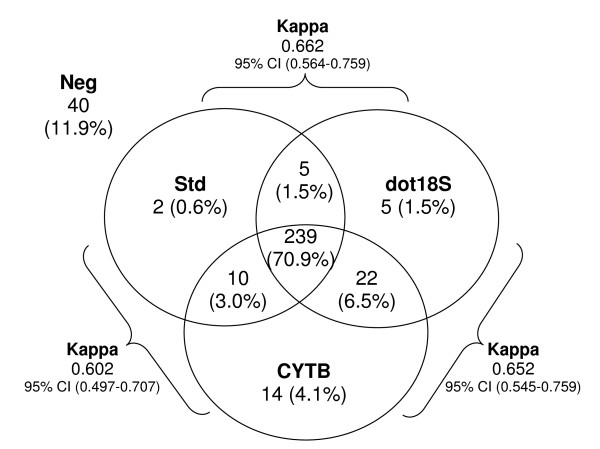
**Agreement of *Plasmodium spp*. detection by the three molecular methods**. The *cytochrome b*-based detection method (CYTB) showed the highest number of *Plasmodium *infections (285 samples), while 271 and 256 infections were observed with the Dot-blot detection (dot18S) and the "standard" nested PCR (Std), respectively. The three methods agreed on the detection of 239 positive and 40 negative samples (agreement rate of 82.8%). Kappa coefficient (Kappa) is calculated between those three methods with 95% Confidence Interval (95% CI).

The sample-by-sample comparison of *Plasmodium spp*. detection showed that the three molecular methods were in agreement for 82.8% of samples, whereas the remainder was detected either by only one (6.2%) or by two methods (11.0%, Figure 3). The dot 18S detection approach displayed a sensitivity of 95.3% and a specificity of 66.7%, while a higher sensitivity (97.3%) and a lower specificity (55.7%) were obtained with the CYTB detection, compared to the "standard" method. These relatively low specificities were due to 27 and 36 "standard" negative samples testing positive with the methods dot18S and CYTB, respectively (Figure 3), most probably because of their higher sensitivities previously shown with serially diluted control samples. In other words they were indeed due to their checking positive samples classified as negative (false negatives) by the "standard" method.

### Comparison of the molecular approaches for species-specific detection

**Figure 4:**
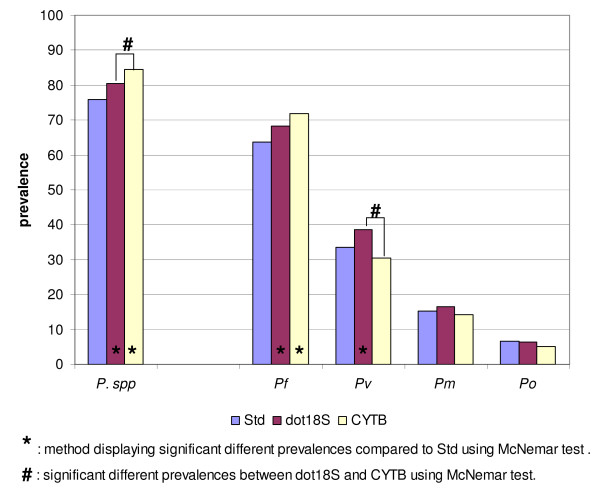
**Genus- and species-specific detection by three molecular methods**. Std = "standard" nested PCR, dot18S = *18S rDNA *based Dot blot detection, CYTB = *cytochrome b *based detection with SNP identification, *P. spp*. = *Plasmodium spp*., *Pf*/*Pv*/*Pm*/*Po *= *P. falciparum*, *P. vivax*, *P. malariae *and *P. ovale*, respectively.

The three molecular methods had different sensitivities for detecting the four *Plasmodium *species (Figure 4). Paired comparison of the prevalence measured by the three methods for *Plasmodium spp *(Std 76.0%, dot18S 80.4%, CYTB 84.6%) and for *P. falciparum *infections (Std 63.8%, dot18S 68.3%, CYTB 71.8%) showed that dot18S and CYTB detected significantly more *Plasmodium spp *(exact McNemar test: Std/dot18S = 0.0237; Std/CYTB = 0.0000; dot18S/CYTB = 0.0243) and *P. falciparum *(exact McNemar test: Std/dot18S = 0.0357; Std/CYTB = 0.0003; dot18S/CYTB = 0.0807) infections than the "standard" method. Significantly higher *P. vivax *prevalence was detected by the dot18S method, compared to the CYTB diagnosis (38.6% versus 30.6%, exact McNemar test: dot18S/CYTB = 0.0009), while the "standard" method measured a prevalence of 33.5% (exact McNemar test: Std/dot18S = 0.0270; Std/CYTB = 0.2888). Comparable prevalences were observed for *P. malariae *(Std 15.1%, dot18S 16.6%, CYTB 14.2%; exact McNemar test: Std/dot18S = 0.4244; Std/CYTB = 0.7493; dot18S/CYTB = 0.2153) and *P. ovale *(Std 6.5%, dot18S 6.2%, CYTB 5.0%; exact McNemar test: Std/dot18S = 1.0000; Std/CYTB = 0.4244; dot18S/CYTB = 0.4807).

With the "standard" and dot18S methods, some samples tested negative with the species-specific detection ("standard": nested PCR, dot18S: hybridization of probes) even though they had been positive with the previous genus-specific nested PCR (Std: 12 samples, dot18S: 7 samples). The comparison of the species identifications sample by sample and species by species showed that agreement of the three molecular methods was best for *P. falciparum*: the three techniques consistently detected *P. falciparum *in 193 samples (see Additional file 1), out of 262 *P. falciparum*-positive samples detected by at least one of the techniques (agreement rate of 73.7%). The agreement rates were much lower for *P. vivax *(42.0%), *P. malariae *(36.8%), and lowest for *P. ovale *(18.4%). The large majority of the samples with no agreement consisted of conflicting detection of *P. malariae *and *P. ovale*.

## Discussion

The WHO gold standard method for malaria diagnosis is microscopy. It is considered inexpensive and field adapted, even though it is time-consuming and requires specifically trained personnel. Molecular detection methods achieve much higher detection sensitivities [1,26], and they are better adapted to automation of the process and objective reading of results by machines. This potential makes them a valuable option for large-scale epidemiologic studies. Unfortunately few efforts have been spent on developing the high-throughput approaches needed for such studies.

The *18S rRNA *gene is the most frequently cited marker for malaria detection. It is composed of highly conserved regions which can be targeted for a qualitative detection of *Plasmodium spp*., and of variable zones allowing species identification [4-7]. However, the *18S rRNA *genes in *Plasmodium spp*. also have unusual properties, such as the existence of three stage-specific A-, S- and O-types, as well as copy number and strain-specific sequence variations [27-29]. Primers and probes have to be designed accordingly, since strain-specific variations can perturb the detection [30,31]. The copy number of the *rRNA *gene varies from four to eight [28,29]. The design of primers and probes for the dot18S method was based on published sequences alignment, by targeting the Zone V7 which displays maximal inter-species sequence variation, while allowing genus-specific identification as well as detection of the *18S rRNA *A- and S- type genes of *P.falciparum *and *P.vivax*. 18S rRNA polymorphism could be a significant biais for molecular when targeting this gene, anyway it is still considered as the "gold standard" for species identification. The *cytochrome b *gene is highly conserved and has mainly been used for phylogenetic studies [23,32-35]. The gene is located on the mitochondrial genome, which occurs at an estimated number of 30–100 copies per parasite [36-38], and as a consequence is predicted to permit more sensitive detection than the *18S rRNA *gene.

Analysis of more than 300 field samples collected in Cambodia demonstrated the added value of the molecular approach. Compared to microscopic diagnosis, an up to two-fold higher *Plasmodium *prevalence was evidenced, revealing a previously unsuspected reservoir of asymptomatic infections. Mixed infections were detected in about one third of the villagers, a much higher figure than reported in neighbouring countries [7,10,11]. Such information is crucial for the National Malaria Control Programme, as mixed infections could influence gametocyte carriage and clinical outcome [14,15,39].

The performance of the two new molecular detection approaches outranged the published "standard" nested PCR method. The *cytochrome b*-based CYTB method was the most sensitive, likely because it targets the multiple copies of mitochondrial genome present in each parasite. Probably for the same reason, it also detected higher rates of *Plasmodium spp*. and *P. falciparum *infections among the field samples. *Plasmodium vivax *was better identified with the dot18S detection approach, maybe because two independent experiments with the A-type and S-type specific probes were combined, increasing the chance of detecting low parasite densities in *P. vivax *infections. The two 18S rDNA-based methods (std and dot18S) detected some samples by genus-specific but not by species-specific amplification/probe hybridization. The reason for this is uncertain but could be due to mutations in the targeted *18S rDNA *sequences, or, in the case of the "standard" method, to copy number variations of the stage-specific gene types amplified by the "standard" method (*P. falciparum*: S-type, *P. vivax*: A-type), as previously reported [28,30,31].

The three molecular methods agreed well on the detection of *Plasmodium spp*. infections. However, the different species were not detected/identified with the same level of agreement, with most disagreements being observed for *P. malariae *and *P. ovale*. This is most probably due to the previously described "all-or-none" effect occurring at threshold parasite densities. Low-levels parasitaemias of *P. malariae *and *P. ovale *are especially frequent in mixed infections, partly because of the species characteristics, partly because of a density-dependant regulation mechanism [40]. These species are, therefore, easily overlooked by microscopy, when *P. falciparum *or *P. vivax *are the major species. This also explains the largely higher number of mixed infection detected with molecular biology tools compared to microscopic examinations.

The weak point of the newly developed molecular techniques remains the visual interpretation of spots with a certain background (dot18S) and of potentially double electrophoresis peaks (CYTB). This may be solved by using straightforward bioinformatics software for analysis. Another weak point could be non-detection of parasites by molecular methods while detected by microscopy, which occurred in a number of samples in this study. The non-detection by molecular methods was most probably due to errors of DNA extraction (false negatives) or error on slide reading (false positives), especially for four samples, which were negative by all three molecular diagnosis methods. Non-detection because of atypical mutations in primer- and/or probes hybridization zones could be a third explanation for these results.

New large-scale detection methods should obviously be evaluated with numerous field samples from different endemic areas of the world, to assess their robustness with respect to possible sequence variations in wild parasites. This is particularly important for the more variable 18S rRNA marker as previous reports indicate that *Plasmodium *parasites from South-East Asia, including North-East Cambodia, display a particularly large heterogeneity of 18S rRNA gene sequences (S. Incardona et al, unpublished work) [24,30,31]. Furthermore, infections with low parasite densities and mixed species, including *P. malariae *and *P. ovale*, are common in this part of the world (S. Incardona et al, unpublished work) [11,14]. It is therefore important to note that this study, with its promising results, provides a field evaluation of the new detection methods in a relatively difficult context.

Even if the two newly developed techniques allow detection of *Plasmodium *genus and species at a much larger scale than the standard molecular diagnosis methods, the ultimate goal of this work is to develop new approaches allowing analysis of thousands of field samples. Many phases of the current processes are amenable to adaptation to pipetting robots, reducing contamination risks, manipulation errors and, therefore, false positive and false negative results. In particular, DNA extraction by pipetting robots and detection with the real-time amplification approach help to reduce the number of manipulation steps and opening of tubes, while allowing to deal with a higher number of samples. Feasibility studies with reference DNA samples and the above described field samples have shown that the dot18S detection approach, using the same primers and probes, could be transferred to a microarray format (S. Incardona et al, unpublished work). Similarly, higher throughput detection of the *cytochrome b *PCR products by RFLP/dHPLC analysis or by microarray has successfully been evaluated (N. Steenkeste et al, unpublished work). These higher throughput approaches should allow *Plasmodium *detection and species identification of thousands of samples in mass screening programme in less than three days of lab work (N. Steenkeste et al, unpublished work).

## Conclusion

This work is a first step on the way to developing high-throughput parasite detection approaches for large-scale field studies. Highly sensitive detection of malaria parasites can be achieved. Sensitive molecular methods will prove useful in studies exploring malaria epidemiology, risk factors for symptomatic and asymptomatic infections, interaction of species and importantly in monitoring efficacy and effectiveness of the scaling up of malaria control efforts. Indeed, classical control measures and symptom-based treatment are inefficient in controlling asymptomatic carriers [13,33,41], yet controlling asymptomatic infections and targeting this reservoir are critical arms of malaria elimination programmes [42].

## Competing interests

The authors declare that they have no competing interests.

## Authors' contributions

NS, LD and FA conceived and designed the genus-specific nested PCR and SNP identification based on the *cytochrome b *gene; SI, FA and TF conceived and designed the genus-specific nested PCR and species-specific dot blot detection based on the *18S rDNA *gene; MTE sequenced the *cytochrome b *PCR products; SH, TS and SD lead the field work; NS, SI, SC managed the experimental procedure and performed the laboratory work; NS, SI, CR, TF, FA participated in the statistical analyses; NS, SI, LD, MTE, PL, SH, TS, SD, CR, OMP, TF, FA drafted and critically revised the manuscript. All authors read and approved manuscript.

## Supplementary Material

Additional File 1 **Agreement of *Plasmodium falciparum *detection by the three molecular methods**. Std = "standard" nested PCR, dot18S = *18S rDNA *based Dot blot detection, CYTB = *cytochrome b *based detection with SNP identification. Kappa coefficient (Kappa) is calculated between those three methods with 95% Confidence Interval (95% CI). Click here for file
