# Supplementary material for: Towards high-throughput molecular detection of Plasmodium: new approaches and molecular markers
Source: Malar J. 2009 Apr 29;8:86. doi: 10.1186/1475-2875-8-86 (PMC2686730; doi:10.1186/1475-2875-8-86)
Supplement: Additional File 1 — Agreement of Plasmodium falciparum detection by the three molecular methods. Std = "standard" nested PCR, dot18S = 18S rDNA based Dot blot detection, CYTB = cytochrome b based detection with SNP identification. Kappa coefficient (Kappa) is calculated between those three methods with 95% Confidence Interval (95% CI). [file 1475-2875-8-86-S1.doc]

**Additional figure 1:**

**Neg**

75 (22.2%)

**Std**

6 (1.8%)

**CYTB**

17 (5.0%)

**dot18S**

7 (2.1%)

7 (2.1%)

193 (57.3%)

9

(2.7%)

23 (6.8%)

**Kappa**

0.703

95% CI (0.623-0.783)

**Kappa**

0.718

95% CI (0.636-0.799)

**Kappa**

0.642

95% CI (0.556-0729)
